# Systemic autoinflammation with intractable epilepsy managed with interleukin-1 blockade

**DOI:** 10.1186/s12974-018-1063-2

**Published:** 2018-02-09

**Authors:** Allen D. DeSena, Thuy Do, Grant S. Schulert

**Affiliations:** 1 Division of Neurology, Department of Pediatrics, Cincinnati Children’s Hospital Medical Center, University of Cincinnati College of Medicine, 3333 Burnet Ave, MLC 2015, Cincinnati, OH 45229 USA; 2 Division of Rheumatology, Department of Pediatrics, Children’s Hospital Medical Center, University of Cincinnati College of Medicine, 3333 Burnet Ave, MLC 4010, Cincinnati, OH 45229 USA

**Keywords:** Seizures, IL-1beta, Anakinra, Canakinumab

## Abstract

**Background:**

Autoinflammatory disorders are distinguished by seemingly random episodes of systemic hyperinflammation, driven in particular by IL-1. Recent pre-clinical work has shown a key role for IL-1 in epilepsy in animal models, and therapies for autoinflammation including IL-1 blockade are proposed for refractory epilepsy.

**Case presentation:**

Here, we report an adolescent female with signs of persistent systemic inflammation and epilepsy unresponsive to multiple anti-epileptic drugs (AED). She was diagnosed with generalized epilepsy with a normal brain MRI and an electroencephalogram (EEG) showing occasional generalized spike and slow wave discharges. Her diagnostic evaluation showed no signs of autoimmunity or genetic causes of epilepsy or periodic fever syndromes but persistently elevated serum inflammatory markers including S100 alarmin proteins. She experienced prompt clinical response to IL-1 blockade with first anakinra and then canakinumab, with near complete resolution of clinical seizures. Additionally, she displayed marked improvements in quality of life and social/academic functioning. Baseline gene expression studies on peripheral blood mononuclear cells (PBMC) from this patient showed significantly activated gene pathways suggesting systemic immune activation, including focal adhesion, platelet activation, and Rap1 signaling, which is an upstream regulator of IL-1β production by the NLRP3 inflammasome. It also showed activation of genes that characterize inflammasome-mediated autoinflammatory disorders and no signs of interferon activation. This gene expression signature was largely extinguished after anakinra treatment.

**Conclusions:**

Together, these findings suggest that patients with epilepsy responsive to immune modulation may have distinct autoinflammatory features supporting IL-1 blockade. As such, IL-1 blockade may be highly efficacious adjunctive medication for certain refractory epilepsy syndromes.

**Electronic supplementary material:**

The online version of this article (10.1186/s12974-018-1063-2) contains supplementary material, which is available to authorized users.

## Background

Autoinflammatory disorders represent a heterogeneous collection of both monogenic and complex diseases, characterized by seemingly random or unprovoked inflammation [1]. In contrast to classic autoimmune diseases, AID typically lack high-titer autoantibodies or autoreactive lymphocytes but are rather characterized by defects in innate immune responses. The best characterized autoinflammatory disorders have been linked to defects in specific pattern recognition receptors in the inflammasome complex, leading to hyperproduction of proinflammatory cytokines particularly of the IL-1 family [2]. These include the cyropyrin-associated periodic syndromes (CAPS), with mutations in NLPR3, which encompasses a broad phenotypic spectrum, from cold-associated urticaria (FCAS) to disorders with significant neurologic involvement and epilepsy such as neonatal-onset multisystem inflammatory disease (NOMID) [3, 4]. Recent work has also highlighted a distinct family of autoinflammatory disorders with prominent over-activation of the type I interferon response including Aicardi-Goutieres syndrome, which causes progressive encephalopathy [5, 6].

Inflammation and particularly pro-inflammatory cytokines such as IL-1 are also increasingly implicated in the pathogenesis of seizures and epilepsy [7, 8]. Indeed, in multiple distinct animal models of epileptogenesis, pharmacologic blockade of IL-1 through the use of the IL-1 receptor antagonist (IL-1RA) reduced seizures and signs of cellular injury [9–11], leading to the proposal that medications for peripheral autoinflammation could be beneficial for intractable epilepsy [12]. Here, we report an adolescent female with signs of systemic inflammation and epilepsy unresponsive to multiple anti-epileptic drugs (AED), with a profound clinical improvement in response to IL-1 blockade. Gene expression profiling demonstrated an inflammatory signature that is largely extinguished upon treatment, further suggesting immune correlates that could identify patients with epilepsy who could benefit from IL-1 blockade.

### Patient data and study approval

This study was approved by the Cincinnati Children’s Hospital Institutional Review Board (IRB 2011-1517), and informed consent was obtained from all patients and/or their legal guardians. Data pertaining to this patient’s clinical course, laboratory values, and treatment were collected from the electronic medical records. Peripheral blood mononuclear cells (PBMC) were obtained and RNA extracted as described [13].

### AmpliSeq transcriptome analysis

Gene expression profiles from PBMC of the patient and healthy controls were determined using the AmpliSeq Transcriptome Gene Expression Kit via the Ion Torrent S5 system (Thermo Fisher, Carlsbad, CA). Differential gene expression was visualized using the Morpheus platform and pathway analysis performed using DAVID Functional Annotation Tool. Full methodologic details are provided in the supplementary information.

## Case presentation

**Fig. 1:**
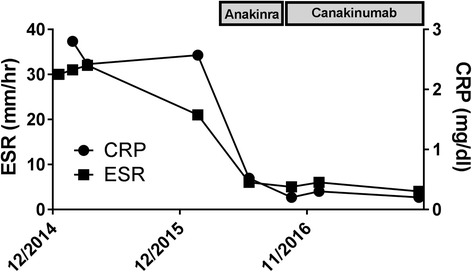
IL-1 blockade leading to resolution of systemic inflammation in patient with refractory epilepsy

The patient is a 14-year-old female diagnosed with generalized epilepsy after presenting with early morning involuntary jerking of her upper extremities and an electroencephalogram (EEG) with occasional generalized spike and slow wave discharges. She later had frequent staring spells, suspected to be absence seizures, along with increasing memory loss and poor academic performance. Notably, she had no history of fevers, rash, arthritis, or serositis. Her brain MRI was normal aside from a single nonspecific dorsal thalamic T2 lesion that has remained stable. Over 2 years, she seized on average 4–15 times daily despite multiple AED. Upon presentation to our neuroimmunology clinic, she was treated with levetiracetam 1500 mg twice daily (later increased to 2000 mg twice daily), ethosuximide 250 mg in the morning and 500 mg in the evening, lamotrigine 100 mg twice daily, and topiramate 100 mg twice daily; clonazepam 0.25 mg was later added also without dramatic reduction in seizure frequency. An extensive laboratory evaluation is summarized in Table 1. Notably, she had negative testing for all autoantibodies but had persistently elevated C-reactive protein and erythrocyte sedimentation rate (ESR) (Fig. 1). Serum levels of S100A8/A9 and S100A12 alarmin proteins, which serve to amplify innate immune responses and inflammation, were also elevated (Table 1) [14]. Both epiSeek Infancy and Childhood Epilepsy Panel and Genetic Periodic Fever Syndromes Panel including *NLRP3* was negative. Cerebral spinal fluid studies showed no elevation in her white blood cells or protein, absent oligoclonal bands, and a normal IgG index and synthesis rate. Cytokine levels in the CSF showed only a mild elevation in IL-1β (25 pg/mL; normal ≤ 10 pg/mL); however, it is unknown how CSF sample processing could affect IL-1 levels. Serum IL-1β was within normal limits, but circulating IL-1 levels are frequently normal even in active systemic autoinflammatory disorders [15]. Finally, a PET scan showed no areas consistent with inflammatory foci.

**Table 1:** Summary of diagnostic evaluation

Test	Results	Normal range
White blood cell count (10^3^/μL)	13.1	4.5–13.0
Absolute neutrophil count (10^3^/μL)	10.2	1.8–8.0
Hemoglobin (g/dL)	13.1	12.0–16.0
Platelet count (10^3^/μL)	300	135–466
AST (U/L)	9	5–26
ALT (U/L)	19	12–49
Albumin (g/dL)	3.7	3.3–4.8
Total protein (g/dL)	7.8	6.4–8.3
TSH (mcIU/mL)	1.39	0.43–4.00
S100A8/A9 (ng/mL)	5617	716–3004
S100A12 (ng/mL)	429	32–385
Erythrocyte sedimentation rate (mm/h)	30	0–20
C-reactive protein (mg/dL)	2.8	< 0.30
IgA (mg/dL)	118	68–376
IgG (mg/dL)	1050	724–1611
IgM (mg/dL)	66	60–264
Anti-nuclear antibody	Negative	< 1:80
Extractable nuclear antigens (Jo-1, Ro, La, RNP, Sm)	Negative	
Anti-dsDNA	Negative	
Anti-phospholipid antibody panel	Negative	
c-ANCA (U/mL)	0	0–19
p-ANCA (U/mL)	0	0–19
Anti-ASMA	Negative	
Anti-LKM	Negative	
Endomysial antibody	< 1:10	< 1:10
Anti-thyroglobulin antibody (U/mL)	10.1	10–114
Anti-thyroid peroxidase antibody (U/mL)	6.6	5–33
Anti-ribosomal antibody (U/mL)	0	0–40
Intrinsic factor blocking antibody	Negative	
Anti-NMO antibody	Negative	
Anti-NMDA receptor ab	Negative	
Paraneoplastic panel	Negative	
CSF RBC count (per mm^3^)	29	0–4
CSF WBC count (per mm^3^)	1	0–4
CSF protein (mg/dL)	37	15–45
CSF glucose (mg/dL)	43	40–70
CSF oligoclonal bands	0	0–4
CSF index	0.39	0.3–0.77

Due to a suspected systemic inflammatory process related to her epilepsy, she was empirically treated with oral dexamethasone 120 mg daily for 5 days, leading to a dramatic but transient response, with no seizures for 1 week followed by regression to her baseline seizure frequency. After a lengthy discussion, treatment with the recombinant IL-1RA anakinra 100 mg daily was initiated, upon which she experienced a rapid approximately 80% reduction in seizure frequency to about four per week. She had rapid normalization in her inflammatory markers (Fig. 1). Anakinra was later increased to 100 mg twice daily, resulting in 2 months without clinically evident seizures. She also noted profound improvements in her fatigue, general malaise, quality of life, and academic performance, now allowing her to work and consider pursuing higher education. Repeat EEG testing showed occasional generalized spike and slow wave discharges with increased frequency in sleep. Repeat CSF examination was not performed. The patient was changed to the anti-IL-1β monoclonal antibody canakinumab 300 mg every 4 weeks, and she enjoyed long periods of being seizure-free, currently averaging one seizure per several months. She was weaned off lamotrigine and ethosuximide, and her clonazepam changed to clobazam.

### Peripheral blood gene expression profiles demonstrate features of autoinflammation

**Fig. 2:**
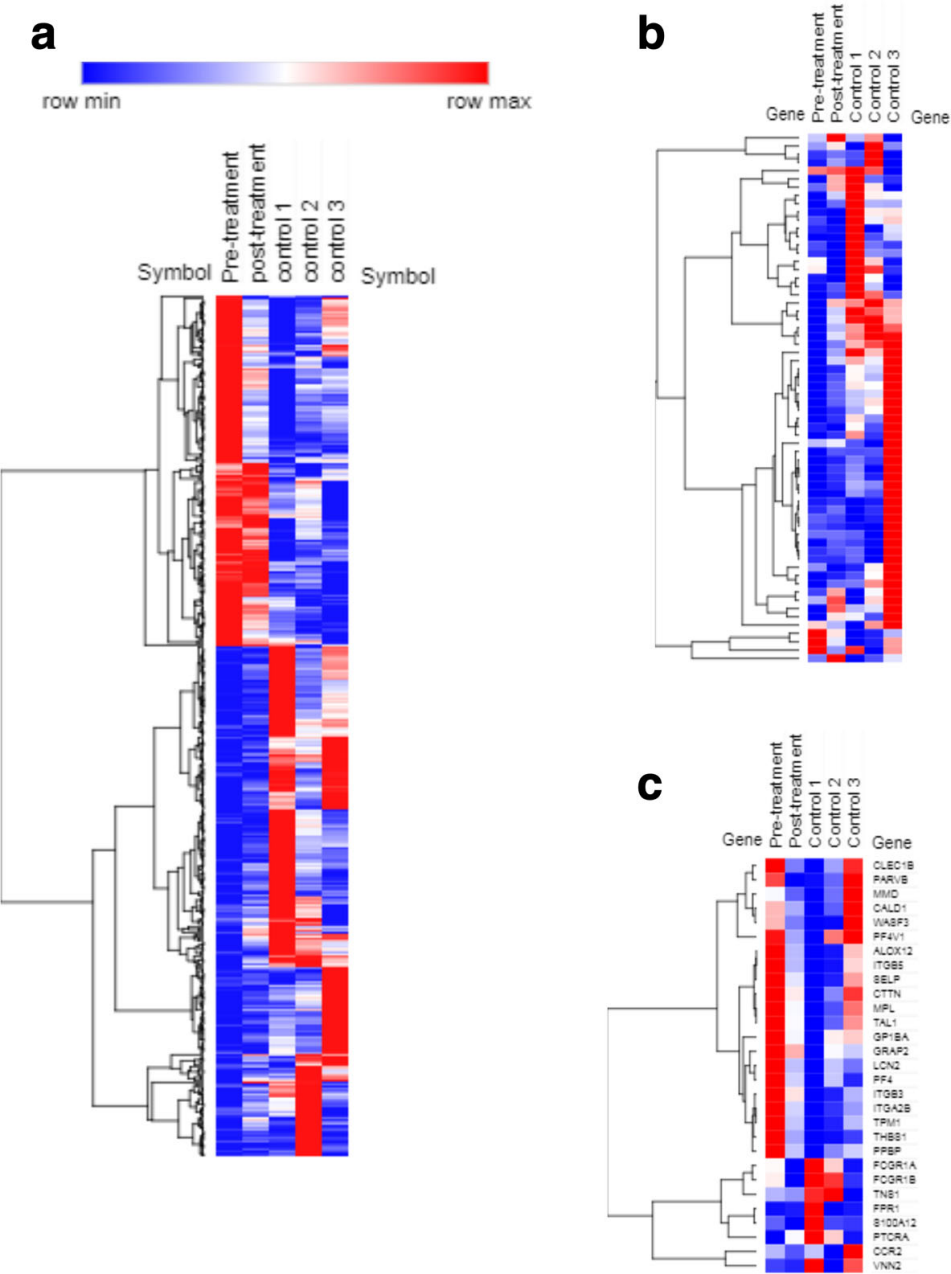
PBMC gene expression signatures before and after anakinra treatment. Gene expression was quantified using the AmpliSeq Transcriptome kit and the Ion Torrent S5 system as described. Heatmaps show hierarchical clustering of normalized log-2 RPKM from patient PBMC as well as three pediatric control samples. **a** Heatmap showing genes with > 2-fold difference between pre-treatment sample and mean of control samples. **b** Heatmap showing genes associated with autoinflammatory interferonopathies, as determined in [6]. **c** Heatmap showing immune response genes associated with the IL-1-driven autoinflammatory disorder CAPS [18]

In order to define the systemic immune activation in this patient, PBMC gene expression profiles were determined both before and after initiation of anakinra treatment and compared to three healthy, age-matched controls using AmpliSeq Transcriptome [16]. Full sequencing details are shown in the supplemental information. Compared to the mean of healthy controls, pre-treatment patient PBMC showed 178 genes upregulated and 260 genes downregulated > 2-fold (Fig. 2a, Additional file 1: Table S3). Pathway analysis of the upregulated genes showed multiple significantly enriched gene pathways suggesting systemic immune activation, including focal adhesion (*p* = 9.1 × 10^−5^), platelet activation (*p* = 0.0011), Rap1 signaling (*p* = 0.0028), and cytokine-cytokine receptor interaction (*p* = 0.018). Of particular note, Rap1 signaling is an upstream regulator of IL-1β production and plays a key role in NLRP3 inflammasome activation [17]. In addition, 83% (148/178) of upregulated genes showed lower expression in patient PBMC after anakinra treatment, including 87.5% (7/8) of elevated Rap1 pathway genes (Additional file 1: Table S4). Regarding downregulated genes, the most significantly enriched GO term was type I interferon signaling pathways (*p* = 8.8 × 10^−8^), strongly supporting the absence of an interferon-induced gene signature in this patient. Indeed, full analysis of the interferon-induced signature described in autoinflammatory interferonopathies [6] shows no evidence of interferon activation before or after anakinra treatment (Fig. 2b).

To further examine for the presence of an autoinflammatory signature responsive to IL-1 blockade, we utilized well-characterized PBMC gene expression profiles that could clearly distinguish patients with CAPS, regardless of underlying disease activity [18]. As shown in Fig. 2c, this patient showed increased expression of a broad spectrum of immune response genes activated in CAPS patients, expression of which was largely extinguished with anakinra treatment. Taken together, these findings suggest that this patient displays features of IL-1-driven systemic autoinflammation, which were markedly reduced with anakinra treatment.

## Discussion and conclusion

There is increasing evidence from animal models that inflammation, and in particular IL-1, is a key driver of epilepsy [7, 9–11]. IL-1 family cytokines are recognized as central to the pathogenesis of systemic autoinflammatory disorders such as CAPS, and blockade of IL-1 using anakinra or canakinumab leads to rapid and sustained clinical improvement in inflammatory symptoms [19]. Here, we describe a patient with refractory epilepsy and features of systemic inflammation including elevated CRP, ESR, and S100 proteins, treated with IL-1 blockade. This patient showed a profound improvement in her symptoms likely related to a systemic inflammatory syndrome and a greater than 90% reduction in seizure frequency since starting anti-IL-1 immunotherapy. Although other changes were made to her AED regimen, sustained seizure reduction seemed to correlate best with initiation of anakinra or canakinumab. In conjunction with seizure reduction, the patient showed sustained normalization of inflammatory markers and marked improvement in quality of life and social/academic functioning.

Although genetic testing for well characterized, monogenetic autoinflammatory disorders was negative, this patient had significant signs of systemic autoinflammation. Her ESR and CRP, while nonspecific, were persistently elevated and normalized in conjunction with seizure control. S100 proteins, which are also associated with systemic autoinflammation [14], were also elevated. She had no features of autoimmunity such as elevated total IgG or specific autoantibodies (Table 1). Most interestingly, PBMC gene expression profiling showed upregulation of numerous gene pathways suggesting systemic immune activation, including upstream regulators of the NLRP3 inflammasome [17], and genes associated with anakinra response [18] (Fig. 2). Indeed, the vast majority of this gene signature was diminished upon anakinra treatment. This suggests that patients with epilepsy responsive to immune modulation may have distinct inflammatory features supporting IL-1 blockade.

The potential utility of IL-1 blockade for seizures has been noted in multiple preclinical studies [7, 8] but to our knowledge has not been reported in patients with idiopathic epilepsy. There is one recent report of a patient with febrile infection-related epilepsy syndrome (FIRES), a rare but devastating encephalopathy occurring after a febrile illness, that had improvement with anakinra while in super-refractory status epilepticus [20]. The patient reported here thus represents the second patient with epilepsy treated with IL-1 blockade (and first treated non-emergently without status epilepticus), suggesting that in selected cases, this treatment might be a profoundly impactful adjunctive medication for certain refractory epilepsy syndromes.

## Additional file

Additional file 1: Supplemental methods; Tables S1–S4. Supplemental methods on gene expression analysis; tables of mapped reads and coverage, genes with > 2-fold difference between pre-treatment sample and controls, and Rap1 pathway genes. (DOCX 51 kb)
