# Supplementary material for: Systemic autoinflammation with intractable epilepsy managed with interleukin-1 blockade
Source: J Neuroinflammation. 2018 Feb 9;15:38. doi: 10.1186/s12974-018-1063-2 (PMC5807745; doi:10.1186/s12974-018-1063-2)
Supplement: Supplementary file 1 — Supplemental methods; Tables S1–S4. Supplemental methods on gene expression analysis; tables of mapped reads and coverage, genes with > 2-fold difference between pre-treatment sample and controls, and Rap1 pathway genes. (DOCX 51 kb) [file 12974_2018_1063_MOESM1_ESM.docx]

**Supplemental Methods**

Total RNA was extracted using the MagMax -96 Total RNA Isolation Kit (Life Technologies), and quantified via Qubit RNA HS Assay Kit (Life Technologies). Using the SuperScript VILO cDNA Synthesis Kit (Life Technologies), 10 ng of RNA was reverse transcribed to make cDNA. The Ion AmpliSeq Library Kit Plus (Life Technologies) and the Ion AmpliSeq Transcriptome Human Gene Expression Core Panel was used to amplify target genes per manufacturer’s directions. Afterward, the FuPa reagent was added to amplicons to partially digest the primers and each sample was barcoded with the Ion Express Barcode Adapters Kit and DNA ligase. Each library was then purified via Agencourt AMPure XP Reagent (Beckman Coulter) and freshly prepared 70% ethanol per manufacture instruction. Subsequently, the purified library was amplified using 1X Library Amp Mix and 25X Library Amp Primers and size selected via Agencourt AMPure Magnetic Beads. Finally, amplified libraries were analyzed with High Sensitivity NGS Fragment Analysis Kit (AATI). All libraries were peaked around 200 bp

Before sequencing, the concentration of the library was determined via Qubit dsDNA HS kit (Life Tech) and diluted with nuclease-free water to 100 pM. Using the Ion 540 Kit-OT2 along with the Ion OneTouch 2 Instrument, the library was amplified on the Ion Spheres Particles (ISP) through emulsion PCR. Afterwards, template-positive ISPs was recovered and the quality of these ISPs was assessed on the Qubit 2.0 Fluorometer with Ion Sphere Quality Control Kit. The Ion One Touch ES and the Dynabeads MyOne Streptavidin C1 Beads (Life Tech) were used to collect the ISPs beads that contained clonally amplified DNA. Using the Ion 540 Kit-OT2, sequencing primers were annealed to the enriched ISPs and loaded on the Ion 540 Chip along with the Ion S5 Sequencing Polymerase. The loaded chip was run on the Ion S5 sequencer, and the amplicon regions were mapped with hg19_AmpliSeq_Transcriptome_ERCC_V1 reference from Ion Community. Full read statistics including mapping are shown in supplemental table 1.

After reads were mapped they were converted into reads per kilobase of transcript per million mapped reads (RPKM). Full mapping statistics for patient and control samples are shown in Supplemental table 2. Genes where the mean RPKM for controls or for pretreated patient sample were less than 5 were excluded from further analysis.

Table S1: Barcode summary report showing total mapped reads, on-target reads, and percent of amplified targets detected for each sample.

| Sample Name | Mapped Reads | On Target | Targets Detected |
| --- | --- | --- | --- |
| Pretreatment (active) | 17,210,685 | 89.38% | 60.98% |
| Posttreatment (inactive) | 18,696,351 | 87.41% | 66.23% |
| PBMCs Healthy 1 | 10,945,889 | 89.77% | 57.32% |
| PBMCs Healthy 2 | 14,348,952 | 83.45% | 66.19% |
| PBMCs Healthy 3 | 23,442,219 | 87.46% | 62.61% |

Table S2: Read coverage showing number of amplified targets for each sample with minimum threshold of mapped reads.

| Sample name | >1 read | >10 reads | >100 reads | >1000 reads | >10,000 reads |
| --- | --- | --- | --- | --- | --- |
| Pretreatment (active) | 15,231 | 12,691 | 9,349 | 2,782 | 186 |
| Posttreatment (inactive) | 16,781 | 13,784 | 9,612 | 3,049 | 191 |
| PBMCs Healthy 1 | 14,643 | 11,930 | 8,081 | 1.793 | 136 |
| PBMCs Healthy 2 | 17,211 | 13,776 | 9,211 | 2,124 | 146 |
| PBMCs Healthy 3 | 16,144 | 13,030 | 9.812 | 3,898 | 259 |

Table S3: Genes showing >2-fold difference between pre-treatment patient sample and mean of controls, in RPKM

| Symbol | Pre-treatment | post-treatment | control 1 | control 2 | control 3 | Change vs. control mean |
| --- | --- | --- | --- | --- | --- | --- |
| AAED1 | 11.831 | 14.992 | 18.928 | 23.051 | 31.459 | 0.483306 |
| ABLIM1 | 12.806 | 6.303 | 5.292 | 2.171 | 8.779 | 2.365349 |
| ACER3 | 9.296 | 13.218 | 23.406 | 11.692 | 28.581 | 0.437947 |
| ACPP | 14.301 | 18.848 | 49.56 | 22.716 | 37.799 | 0.389762 |
| ACVR2A | 5.33 | 7.098 | 8.955 | 8.352 | 16.924 | 0.46712 |
| ADCY3 | 33.607 | 33.167 | 14.959 | 16.703 | 18.046 | 2.028265 |
| ADORA2A | 17.031 | 21.234 | 6.309 | 12.11 | 7.072 | 2.004354 |
| ADRB2 | 17.421 | 23.315 | 12.619 | 25.723 | 67.307 | 0.494685 |
| AGPAT9 | 21.126 | 34.88 | 66.961 | 34.409 | 27.654 | 0.491211 |
| AHNAK | 73.975 | 95.4 | 166.997 | 104.98 | 218.602 | 0.452374 |
| AHR | 18.201 | 14.992 | 53.834 | 19.543 | 53.016 | 0.43201 |
| AIM2 | 7.085 | 6.609 | 22.999 | 15.451 | 15.656 | 0.39284 |
| ALDH1A1 | 20.866 | 32.432 | 86.602 | 38.084 | 28.093 | 0.409729 |
| ALDH3B1 | 14.236 | 19.949 | 58.82 | 18.374 | 11.559 | 0.481201 |
| ALOX12 | 35.947 | 16.461 | 5.292 | 6.431 | 24.289 | 2.994585 |
| ANKRD20A9P | 29.122 | 83.957 | 29.817 | 119.93 | 32.385 | 0.479685 |
| ANPEP | 52.979 | 65.049 | 213.605 | 125.275 | 56.284 | 0.402205 |
| ANXA1 | 444.175 | 412.015 | 1277.661 | 718.576 | 939.908 | 0.453835 |
| ANXA4 | 23.857 | 28.883 | 61.873 | 49.358 | 44.384 | 0.459924 |
| ANXA5 | 96.792 | 122.57 | 268.558 | 96.295 | 239.623 | 0.480376 |
| AP1S2 | 271.458 | 285.528 | 789.901 | 355.112 | 694.433 | 0.442728 |
| APBB1 | 70.335 | 72.636 | 31.344 | 35.327 | 18.583 | 2.475016 |
| APOBEC3A | 28.017 | 24.783 | 94.54 | 40.422 | 75.842 | 0.398716 |
| AQP9 | 24.962 | 19.398 | 56.581 | 33.574 | 61.259 | 0.494578 |
| ARHGAP10 | 24.897 | 15.421 | 11.398 | 8.936 | 10.096 | 2.454519 |
| ARHGAP6 | 16.121 | 6.058 | 1.934 | 5.095 | 12.486 | 2.478248 |
| ARHGEF5 | 12.221 | 14.136 | 6.615 | 6.264 | 2.487 | 2.385982 |
| ARL5A | 23.662 | 32.127 | 37.45 | 35.662 | 71.453 | 0.491032 |
| ARL8A | 23.207 | 56.237 | 87.62 | 46.018 | 46.578 | 0.38632 |
| ARRDC4 | 6.24 | 6.058 | 16.079 | 11.358 | 18.778 | 0.405063 |
| AS3MT | 18.201 | 20.745 | 8.243 | 12.611 | 5.072 | 2.10611 |
| ASH1L-AS1 | 9.556 | 14.564 | 12.721 | 27.644 | 23.753 | 0.447113 |
| ASXL1 | 45.308 | 36.226 | 23.813 | 20.629 | 18.778 | 2.150016 |
| ATP1B3 | 22.492 | 33.044 | 67.674 | 59.38 | 67.551 | 0.346733 |
| AURKAPS1 | 11.701 | 14.809 | 23.101 | 34.576 | 16.29 | 0.474577 |
| BCL11B | 199.889 | 185.049 | 52.307 | 44.013 | 35.848 | 4.537157 |
| BCL6 | 39.328 | 49.016 | 168.421 | 62.804 | 42.677 | 0.430753 |
| BEND2 | 22.492 | 11.81 | 3.867 | 7.6 | 17.461 | 2.33255 |
| BET3L | 17.291 | 7.098 | 4.274 | 5.93 | 7.365 | 2.95253 |
| BLVRA | 42.968 | 61.805 | 112.145 | 110.91 | 91.742 | 0.409483 |
| BRI3 | 82.426 | 102.988 | 298.986 | 242.532 | 90.913 | 0.390996 |
| C10orf11 | 9.556 | 18.786 | 48.542 | 20.127 | 12.827 | 0.351772 |
| C1orf21 | 9.881 | 12.116 | 9.973 | 27.31 | 34.044 | 0.415593 |
| C2orf49 | 23.792 | 28.027 | 52.714 | 47.27 | 59.259 | 0.448221 |
| C4orf21 | 10.986 | 9.791 | 6.208 | 5.345 | 3.463 | 2.194859 |
| C7orf55 | 31.267 | 36.043 | 12.212 | 1.086 | 20.192 | 2.800866 |
| CARD11 | 135.664 | 134.809 | 49.967 | 70.655 | 73.94 | 2.091837 |
| CBFA2T2 | 209.184 | 161.306 | 101.053 | 59.13 | 71.843 | 2.704662 |
| CCDC134 | 119.868 | 121.163 | 56.683 | 42.092 | 23.753 | 2.934872 |
| CCDC144A | 21.646 | 28.7 | 7.836 | 6.18 | 14.242 | 2.298039 |
| CCDC91 | 409.593 | 332.769 | 199.154 | 178.642 | 150.807 | 2.324578 |
| CCNYL1 | 6.5 | 7.955 | 14.756 | 8.185 | 19.704 | 0.457263 |
| CD101 | 21.126 | 23.927 | 88.739 | 25.807 | 37.507 | 0.416815 |
| CD1C | 10.596 | 12.973 | 36.432 | 25.974 | 8.291 | 0.449637 |
| CD300E | 52.329 | 69.699 | 178.903 | 81.262 | 136.955 | 0.395314 |
| CD300LF | 8.581 | 12.055 | 36.635 | 17.706 | 13.608 | 0.378858 |
| CD302 | 76.38 | 73.616 | 282.195 | 95.71 | 197.678 | 0.398101 |
| CD74 | 152.37 | 256.094 | 485.929 | 387.35 | 293.859 | 0.39165 |
| CD86 | 53.499 | 54.34 | 161.807 | 75.165 | 130.029 | 0.43732 |
| CDA | 26.392 | 31.576 | 96.575 | 38.501 | 28.484 | 0.484079 |
| CDC27 | 142.1 | 410.974 | 119.167 | 2928.508 | 227.186 | 0.130173 |
| CDK10 | 67.67 | 76.063 | 40.299 | 45.015 | 15.656 | 2.010597 |
| CDKN1A | 36.988 | 26.741 | 100.747 | 61.468 | 71.209 | 0.475375 |
| CDKN1C | 7.801 | 11.076 | 30.631 | 16.286 | 11.706 | 0.399212 |
| CDYL2 | 6.63 | 8.261 | 14.247 | 12.026 | 21.363 | 0.417541 |
| CEACAM3 | 6.305 | 11.872 | 15.061 | 13.864 | 9.023 | 0.498445 |
| CENPV | 15.016 | 15.421 | 6.004 | 10.189 | 2.634 | 2.392734 |
| CHAMP1 | 10.921 | 12.667 | 7.836 | 9.688 | 57.991 | 0.433861 |
| CKAP4 | 11.311 | 14.442 | 27.477 | 15.701 | 28.191 | 0.475459 |
| CKB | 6.175 | 8.139 | 22.49 | 13.947 | 3.121 | 0.4683 |
| CLEC17A | 17.421 | 11.872 | 4.071 | 6.013 | 10.779 | 2.505057 |
| CLEC4C | 9.426 | 27.721 | 40.197 | 15.785 | 4.78 | 0.46539 |
| CLEC7A | 12.351 | 14.136 | 56.48 | 33.407 | 24.143 | 0.324941 |
| CLINT1 | 33.412 | 59.969 | 56.378 | 52.699 | 117.007 | 0.443357 |
| CLMN | 6.5 | 5.936 | 11.092 | 9.354 | 18.778 | 0.497145 |
| CLU | 247.472 | 143.009 | 26.255 | 57.042 | 63.698 | 5.050621 |
| CMPK2 | 7.085 | 15.665 | 37.043 | 28.479 | 48.139 | 0.187003 |
| CMTM5 | 20.866 | 9.913 | 1.832 | 11.024 | 4.585 | 3.589129 |
| CORO1C | 264.698 | 142.091 | 121.813 | 66.062 | 146.222 | 2.376837 |
| CPNE8 | 10.206 | 13.279 | 29.512 | 13.279 | 23.119 | 0.464543 |
| CPPED1 | 46.803 | 51.464 | 139.418 | 67.982 | 81.744 | 0.485602 |
| CRIP1 | 37.833 | 60.031 | 108.075 | 111.912 | 16.144 | 0.480661 |
| CSRNP1 | 5.135 | 6.67 | 21.778 | 13.363 | 3.658 | 0.397046 |
| CTTN | 32.697 | 20.316 | 5.801 | 12.945 | 29.947 | 2.014478 |
| CUEDC1 | 7.671 | 9.179 | 24.118 | 11.024 | 11.023 | 0.498495 |
| CXCL16 | 7.931 | 10.464 | 29.919 | 18.791 | 21.07 | 0.340972 |
| CXCR2P1 | 195.923 | 102.132 | 42.029 | 111.996 | 122.713 | 2.123919 |
| CYBB | 471.217 | 408.649 | 1367.52 | 669.218 | 1184.701 | 0.438826 |
| CYBRD1 | 33.607 | 32.677 | 104.716 | 52.281 | 92.718 | 0.403744 |
| CYP2S1 | 5.07 | 7.894 | 21.371 | 8.602 | 17.022 | 0.323651 |
| CYSLTR2 | 5.98 | 9.424 | 11.296 | 13.697 | 10.925 | 0.499471 |
| DAPK1 | 38.093 | 43.264 | 145.219 | 65.644 | 57.065 | 0.426529 |
| DHDDS | 102.902 | 71.841 | 41.927 | 25.473 | 29.118 | 3.198429 |
| DKFZP586I1420 | 19.631 | 32.127 | 32.768 | 42.928 | 56.918 | 0.444093 |
| DMXL2 | 59.739 | 49.444 | 148.374 | 81.345 | 168.658 | 0.449868 |
| DNAJB2 | 20.216 | 15.176 | 9.973 | 8.018 | 8.048 | 2.329122 |
| DNAJB7 | 6.305 | 9.363 | 7.327 | 26.057 | 10.145 | 0.434538 |
| DNM3 | 13.846 | 8.445 | 3.562 | 4.76 | 8.974 | 2.401596 |
| DOCK5 | 13.651 | 13.646 | 36.737 | 24.053 | 37.507 | 0.416625 |
| DUSP1 | 293.82 | 166.935 | 879.454 | 1292.753 | 375.261 | 0.346014 |
| DUSP3 | 7.931 | 9.24 | 20.964 | 11.442 | 20.826 | 0.446968 |
| DUSP5 | 11.896 | 16.094 | 37.551 | 20.295 | 26.045 | 0.425409 |
| DYNLL2 | 58.959 | 52.381 | 31.445 | 29.732 | 25.947 | 2.030175 |
| EDAR | 30.682 | 27.415 | 9.769 | 12.11 | 9.072 | 2.973927 |
| EFCAB13 | 37.638 | 27.537 | 0.712 | 15.785 | 38.043 | 2.070297 |
| EGR1 | 39.913 | 1.346 | 13.128 | 7.099 | 2.097 | 5.363689 |
| EIF2AK2 | 67.02 | 77.96 | 111.636 | 88.444 | 253.426 | 0.443346 |
| EIF5A | 300.515 | 344.396 | 394.238 | 555.134 | 894.403 | 0.488967 |
| EMILIN2 | 39.523 | 45.834 | 85.788 | 70.154 | 117.007 | 0.4344 |
| EMR2 | 36.467 | 52.749 | 163.537 | 99.468 | 70.429 | 0.328104 |
| ENTPD1 | 21.841 | 27.721 | 72.762 | 29.899 | 44.286 | 0.445895 |
| ENY2 | 14.951 | 24.722 | 47.931 | 32.07 | 21.948 | 0.439955 |
| EOMES | 31.657 | 35.859 | 12.212 | 36.163 | 198.263 | 0.385062 |
| EPB41L3 | 24.507 | 32.861 | 87.62 | 61.134 | 69.209 | 0.33731 |
| EPB49 | 24.702 | 12.545 | 4.885 | 9.771 | 7.657 | 3.321203 |
| ERBB2 | 12.351 | 16.277 | 5.19 | 5.178 | 7.121 | 2.118646 |
| F13A1 | 442.42 | 216.992 | 155.802 | 107.319 | 373.456 | 2.084995 |
| FABP5 | 14.301 | 24.722 | 27.68 | 42.259 | 18.339 | 0.485999 |
| FAM102B | 12.156 | 16.889 | 20.557 | 14.532 | 41.701 | 0.474906 |
| FAM114A2 | 33.672 | 33.412 | 20.048 | 14.782 | 14.681 | 2.040274 |
| FAM49A | 50.833 | 72.636 | 131.888 | 71.741 | 112.764 | 0.481992 |
| FAM98B | 28.342 | 17.685 | 13.535 | 13.78 | 7.95 | 2.411059 |
| FAR1 | 24.312 | 34.268 | 47.83 | 35.829 | 77.452 | 0.452707 |
| FBXO44 | 18.721 | 15.115 | 6.716 | 12.026 | 7.121 | 2.171558 |
| FBXW4 | 302.206 | 304.07 | 136.06 | 149.411 | 57.65 | 2.642269 |
| FCER1G | 349.919 | 439.062 | 961.681 | 585.2 | 603.373 | 0.488201 |
| FCER2 | 109.077 | 91.362 | 37.348 | 47.604 | 46.481 | 2.489717 |
| FCRL5 | 7.996 | 9.424 | 24.932 | 8.769 | 35.214 | 0.348081 |
| FCRL6 | 44.723 | 69.148 | 39.18 | 127.112 | 135.736 | 0.444227 |
| FFAR2 | 5.265 | 5.079 | 8.955 | 26.141 | 3.512 | 0.409112 |
| FGD6 | 6.825 | 9.424 | 21.574 | 12.026 | 13.218 | 0.437332 |
| FGGY | 194.363 | 138.664 | 115.707 | 80.844 | 39.36 | 2.471648 |
| FGR | 181.947 | 296.971 | 523.989 | 343.337 | 278.544 | 0.476355 |
| FLJ42627 | 18.071 | 27.353 | 41.927 | 71.24 | 10.194 | 0.439466 |
| FLT4 | 13.586 | 10.342 | 2.646 | 7.767 | 7.462 | 2.280168 |
| FMN1 | 18.006 | 12.116 | 30.631 | 104.73 | 68.721 | 0.264688 |
| FOXD2-AS1 | 7.931 | 16.951 | 11.499 | 35.16 | 16.29 | 0.377973 |
| FSTL1 | 22.297 | 8.995 | 1.323 | 2.84 | 12.779 | 3.948235 |
| GAB2 | 7.866 | 9.975 | 27.68 | 12.277 | 17.022 | 0.414153 |
| GIMAP5 | 26.262 | 30.413 | 7.632 | 17.455 | 10.194 | 2.2331 |
| GIPC1 | 21.971 | 22.152 | 10.177 | 12.611 | 8.438 | 2.110837 |
| GLIPR2 | 53.239 | 66.395 | 134.635 | 68.233 | 133.834 | 0.474357 |
| GLT1D1 | 21.516 | 23.498 | 61.263 | 34.659 | 46.188 | 0.454212 |
| GNAZ | 74.69 | 37.022 | 8.854 | 24.554 | 56.187 | 2.500921 |
| GNG11 | 72.415 | 32.738 | 9.464 | 25.974 | 18.534 | 4.025143 |
| GNLY | 765.817 | 1160.899 | 521.75 | 2345.144 | 1735.546 | 0.499181 |
| GNRHR2 | 5.005 | 8.934 | 3.46 | 20.545 | 6.243 | 0.496396 |
| GP6 | 26.392 | 10.036 | 1.221 | 5.679 | 12.096 | 4.168035 |
| GP9 | 111.938 | 48.832 | 19.742 | 43.679 | 31.41 | 3.541184 |
| GPR132 | 24.247 | 22.886 | 9.871 | 9.604 | 9.316 | 2.526519 |
| GPR21 | 9.621 | 19.031 | 12.415 | 32.906 | 14.242 | 0.484579 |
| GPR89C | 21.126 | 13.095 | 7.327 | 7.099 | 9.267 | 2.674967 |
| GRAP | 30.682 | 33.044 | 11.296 | 22.215 | 3.219 | 2.506017 |
| GRAP2 | 318.067 | 236.084 | 81.31 | 197.099 | 176.071 | 2.099545 |
| GSK3A | 50.183 | 128.812 | 194.066 | 125.692 | 180.315 | 0.301054 |
| GTF2IRD2B | 62.534 | 65.722 | 35.72 | 33.991 | 17.022 | 2.162983 |
| GZMB | 64.744 | 127.099 | 147.051 | 131.789 | 140.174 | 0.463545 |
| GZMH | 53.044 | 78.022 | 76.527 | 270.092 | 141.686 | 0.325886 |
| HAUS6 | 95.882 | 113.024 | 0 | 59.38 | 61.844 | 2.372847 |
| HBA2 | 10.791 | 2.509 | 18.827 | 1400.405 | 175.242 | 0.020303 |
| HBB | 8.776 | 0.428 | 11.703 | 1179.838 | 53.212 | 0.021151 |
| HDAC9 | 6.045 | 6.486 | 15.265 | 6.264 | 20.534 | 0.431139 |
| HDHD3 | 6.11 | 7.404 | 9.464 | 11.776 | 16.68 | 0.483386 |
| HERC2P7 | 13.196 | 15.176 | 3.969 | 8.352 | 4.438 | 2.362193 |
| HERC5 | 17.876 | 16.706 | 26.662 | 28.897 | 56.382 | 0.479074 |
| HIP1R | 65.394 | 55.38 | 23.813 | 42.76 | 29.508 | 2.04184 |
| HIST1H1E | 281.989 | 423.703 | 582.707 | 894.295 | 479.685 | 0.432347 |
| HIST1H2AH | 28.667 | 43.692 | 37.958 | 74.831 | 77.159 | 0.452761 |
| HIST1H2AJ | 15.081 | 31.025 | 35.211 | 56.958 | 19.461 | 0.405294 |
| HIST1H2BM | 8.321 | 15.849 | 21.269 | 42.259 | 30.581 | 0.265256 |
| HIST1H4K | 10.531 | 13.952 | 6.92 | 23.051 | 36.677 | 0.474028 |
| HLA-C | 463.872 | 665.416 | 674.805 | 831.825 | 1472.219 | 0.467166 |
| HLA-DMA | 64.614 | 104.641 | 193.557 | 154.756 | 120.47 | 0.4135 |
| HLA-DPA1 | 530.696 | 675.085 | 2719.572 | 1319.895 | 1222.159 | 0.302585 |
| HMGA1 | 30.682 | 1.101 | 0.204 | 0 | 31.751 | 2.880488 |
| HMOX1 | 77.745 | 60.52 | 250.139 | 167.952 | 54.138 | 0.493902 |
| HN1L | 51.288 | 38.001 | 28.8 | 22.967 | 21.85 | 2.090061 |
| HOMER1 | 21.646 | 18.48 | 10.482 | 12.277 | 6.975 | 2.183964 |
| HSPA7 | 38.548 | 56.114 | 142.166 | 94.457 | 23.704 | 0.444226 |
| HSPBP1 | 43.683 | 31.759 | 14.044 | 26.224 | 23.704 | 2.048537 |
| IER5 | 104.397 | 95.645 | 335.723 | 200.273 | 118.812 | 0.478294 |
| IFI44 | 42.903 | 56.237 | 125.069 | 81.93 | 186.46 | 0.327122 |
| IFI44L | 5.72 | 9.546 | 24.118 | 19.793 | 58.625 | 0.167356 |
| IFI6 | 68.58 | 89.036 | 202.716 | 176.22 | 329.951 | 0.29023 |
| IFIT1 | 8.256 | 7.282 | 15.875 | 9.855 | 24.045 | 0.497599 |
| IFIT2 | 23.402 | 38.062 | 63.501 | 60.048 | 96.376 | 0.319227 |
| IGHMBP2 | 13.261 | 13.218 | 4.986 | 5.512 | 7.95 | 2.156494 |
| IGJ | 53.109 | 66.272 | 78.461 | 49.191 | 224.064 | 0.452999 |
| IGSF6 | 35.492 | 46.079 | 102.885 | 50.611 | 76.671 | 0.462603 |
| IL15 | 5.135 | 9.363 | 14.552 | 14.866 | 11.315 | 0.378195 |
| IL4R | 151.33 | 194.901 | 44.268 | 98.299 | 72.623 | 2.109717 |
| IMPAD1 | 45.503 | 50.301 | 80.802 | 55.622 | 142.174 | 0.489986 |
| INPP5F | 5.33 | 6.425 | 6.208 | 8.936 | 19.022 | 0.468009 |
| ISG15 | 15.341 | 19.827 | 49.254 | 42.176 | 21.363 | 0.408031 |
| ITGA2B | 156.336 | 70.862 | 10.38 | 24.721 | 54.724 | 5.221353 |
| ITGB3 | 476.677 | 284.916 | 45.998 | 70.989 | 198.068 | 4.538988 |
| ITGB5 | 26.782 | 12.606 | 4.478 | 5.178 | 17.461 | 2.962938 |
| JUP | 12.611 | 24.661 | 64.519 | 31.319 | 52.48 | 0.25508 |
| KCNQ1OT1 | 119.933 | 267.231 | 113.773 | 434.787 | 176.266 | 0.496394 |
| KCTD3 | 6.5 | 8.751 | 15.977 | 13.864 | 9.462 | 0.496145 |
| KIAA1598 | 20.736 | 16.889 | 99.526 | 18.875 | 32.922 | 0.411094 |
| KIAA1919 | 73.065 | 51.158 | 30.53 | 18.207 | 21.558 | 3.118216 |
| KIFC3 | 14.431 | 10.831 | 4.986 | 7.349 | 8.145 | 2.113916 |
| KLF10 | 72.61 | 72.698 | 393.424 | 182.066 | 168.121 | 0.292935 |
| KLF11 | 58.894 | 58.378 | 189.996 | 119.596 | 80.086 | 0.453405 |
| KLF2 | 153.995 | 122.387 | 54.241 | 45.934 | 125.64 | 2.045856 |
| KLF4 | 48.623 | 36.961 | 179.921 | 110.158 | 56.723 | 0.420612 |
| KLRC3 | 28.732 | 38.98 | 31.242 | 117.341 | 27.654 | 0.489091 |
| KRT72 | 25.547 | 31.392 | 7.429 | 2.84 | 5.511 | 4.856844 |
| KRT73 | 29.707 | 25.273 | 10.278 | 2.673 | 14.388 | 3.259849 |
| LGALS1 | 50.443 | 83.162 | 166.386 | 125.358 | 28.24 | 0.472927 |
| LGALS2 | 23.272 | 42.223 | 94.438 | 40.338 | 28.142 | 0.428535 |
| LGALS9 | 56.294 | 57.216 | 169.643 | 73.244 | 102.619 | 0.488796 |
| LGALSL | 64.809 | 29.434 | 11.703 | 15.284 | 31.898 | 3.301809 |
| LILRA3 | 25.807 | 22.458 | 71.134 | 82.431 | 14.486 | 0.460699 |
| LILRA5 | 28.667 | 17.991 | 96.982 | 54.453 | 26.533 | 0.483239 |
| LILRB2 | 78.525 | 82.856 | 235.892 | 106.484 | 157.586 | 0.471186 |
| LINC00115 | 5.135 | 6.486 | 7.022 | 16.62 | 10.194 | 0.455284 |
| LINC00667 | 5.33 | 2.631 | 10.177 | 7.015 | 17.363 | 0.462741 |
| LOC100132832 | 155.361 | 151.331 | 67.776 | 58.712 | 73.404 | 2.331674 |
| LOC100216545 | 12.416 | 7.772 | 3.256 | 8.686 | 4.975 | 2.201809 |
| LOC100287632 | 5.46 | 8.139 | 10.075 | 26.391 | 2.536 | 0.419978 |
| LOC100506035 | 21.646 | 10.525 | 6.004 | 7.767 | 16.144 | 2.17075 |
| LOC145474 | 14.236 | 22.03 | 44.268 | 37.332 | 11.901 | 0.456765 |
| LOC401321 | 5.33 | 9.913 | 8.65 | 42.259 | 8.096 | 0.270994 |
| LOC729737 | 38.223 | 48.526 | 190.199 | 96.211 | 89.645 | 0.304926 |
| LPCAT2 | 40.238 | 40.388 | 130.259 | 52.699 | 100.717 | 0.425536 |
| LRRC25 | 127.604 | 125.813 | 505.976 | 199.521 | 255.718 | 0.398258 |
| LRRFIP1 | 257.418 | 282.468 | 515.339 | 410.734 | 822.268 | 0.441707 |
| LRRK2 | 57.269 | 57.155 | 194.778 | 71.574 | 131.736 | 0.43158 |
| LTB | 256.572 | 249.485 | 86.399 | 128.532 | 128.274 | 2.24273 |
| LTK | 17.291 | 15.115 | 7.327 | 10.022 | 4.438 | 2.380915 |
| LY6G6E | 19.696 | 10.097 | 2.137 | 7.516 | 8.73 | 3.214274 |
| LY6G6F | 37.183 | 13.646 | 2.849 | 9.103 | 5.853 | 6.265038 |
| LY9 | 52.199 | 46.017 | 20.149 | 23.218 | 24.63 | 2.302999 |
| LYN | 105.047 | 114.615 | 275.376 | 185.908 | 183.631 | 0.488655 |
| LYZ | 2491.035 | 2445.714 | 7620.174 | 6809.438 | 12209.89 | 0.280527 |
| MAN1B1 | 10.791 | 11.994 | 14.959 | 43.178 | 10.779 | 0.469746 |
| MAN2C1 | 16.901 | 23.865 | 8.141 | 5.763 | 8.389 | 2.274391 |
| MAPK13 | 60.519 | 49.567 | 21.065 | 39.67 | 26.972 | 2.07004 |
| MCF2L | 16.511 | 18.052 | 4.783 | 7.349 | 5.268 | 2.846724 |
| MCM9 | 7.671 | 10.525 | 15.774 | 11.692 | 25.703 | 0.432827 |
| MCOLN2 | 6.76 | 12.055 | 8.955 | 19.877 | 21.411 | 0.403638 |
| MDGA1 | 17.096 | 22.948 | 7.124 | 1.253 | 10.194 | 2.761725 |
| ME2 | 12.676 | 13.157 | 28.8 | 17.789 | 43.603 | 0.421634 |
| MED9 | 14.626 | 8.445 | 5.19 | 8.101 | 7.414 | 2.119198 |
| MEGF9 | 98.677 | 93.748 | 279.549 | 108.404 | 286.933 | 0.438639 |
| METTL8 | 40.173 | 23.437 | 21.676 | 10.857 | 14.095 | 2.584692 |
| MFAP3L | 20.541 | 12.116 | 5.292 | 10.022 | 12.047 | 2.25222 |
| MGLL | 20.476 | 10.28 | 6.818 | 8.435 | 13.656 | 2.124875 |
| MIR548I1 | 6.825 | 15.176 | 7.327 | 38.835 | 8.779 | 0.372673 |
| MNDA | 298.435 | 318.389 | 856.964 | 428.69 | 509.778 | 0.498657 |
| MPL | 31.527 | 16.951 | 3.155 | 7.6 | 25.167 | 2.632955 |
| MRPS21 | 5.2 | 6.792 | 7.836 | 123.02 | 28.288 | 0.098024 |
| MRPS26 | 34.192 | 35.125 | 16.486 | 23.134 | 11.364 | 2.011925 |
| MRS2P2 | 8.971 | 9.301 | 18.623 | 31.486 | 4.877 | 0.489452 |
| MS4A14 | 5.785 | 7.588 | 20.251 | 11.609 | 24.923 | 0.305637 |
| MS4A7 | 14.431 | 24.233 | 49.356 | 25.723 | 31.995 | 0.404328 |
| MT2A | 65.459 | 88.363 | 186.027 | 220.484 | 197.629 | 0.325052 |
| MTX2 | 33.542 | 26.986 | 15.061 | 12.695 | 11.315 | 2.575465 |
| MX1 | 70.595 | 89.587 | 147.865 | 187.161 | 302.052 | 0.332432 |
| MX2 | 24.832 | 30.964 | 44.777 | 28.646 | 91.303 | 0.452242 |
| MYADM | 198.394 | 190.617 | 502.72 | 264.831 | 497.243 | 0.470576 |
| MYL9 | 373.775 | 133.096 | 16.181 | 53.033 | 101.985 | 6.549834 |
| NAAA | 56.749 | 70.189 | 164.86 | 79.925 | 142.418 | 0.439684 |
| NAMPT | 48.688 | 56.359 | 191.217 | 86.356 | 134.614 | 0.354363 |
| NANOG | 20.216 | 36.532 | 64.316 | 84.435 | 28.142 | 0.342851 |
| NANOS1 | 6.11 | 10.954 | 9.566 | 16.286 | 14.095 | 0.458858 |
| NAP1L5 | 12.286 | 18.786 | 26.662 | 24.888 | 25.899 | 0.4759 |
| NAPSB | 13.196 | 22.58 | 53.427 | 36.664 | 10.633 | 0.393034 |
| NEXN | 27.887 | 6.976 | 4.376 | 13.196 | 11.559 | 2.871889 |
| NFKBIA | 106.867 | 109.291 | 343.966 | 239.024 | 158.074 | 0.432623 |
| NFRKB | 75.47 | 52.381 | 34.702 | 36.497 | 37.507 | 2.082774 |
| NLK | 114.928 | 68.292 | 55.971 | 47.604 | 62.674 | 2.073901 |
| NPM3 | 22.102 | 18.725 | 11.601 | 14.198 | 5.268 | 2.13429 |
| NRGN | 229.141 | 120.612 | 25.441 | 51.363 | 80.134 | 4.38022 |
| NRIP1 | 821.721 | 418.195 | 277.615 | 150.747 | 137.297 | 4.358037 |
| OAS3 | 46.868 | 57.767 | 125.375 | 58.295 | 156.611 | 0.4132 |
| OBSCN | 48.818 | 38.307 | 20.353 | 31.653 | 10.047 | 2.360144 |
| ODF3B | 7.085 | 13.952 | 35.72 | 38.418 | 17.461 | 0.232044 |
| OGFRL1 | 38.288 | 24.233 | 73.373 | 37.917 | 168.56 | 0.410448 |
| OR52K2 | 7.28 | 12.422 | 19.742 | 21.547 | 13.169 | 0.401043 |
| OSTF1 | 67.28 | 82.733 | 101.562 | 94.958 | 227.82 | 0.475656 |
| PAIP2B | 17.161 | 14.197 | 8.65 | 10.69 | 6.389 | 2.000972 |
| PAK1 | 105.697 | 116.39 | 315.981 | 194.009 | 129.444 | 0.495893 |
| PBX1 | 23.272 | 11.321 | 4.579 | 8.686 | 11.559 | 2.81244 |
| PCSK6 | 41.993 | 19.154 | 3.053 | 9.437 | 12.876 | 4.966451 |
| PDCD6IP | 30.422 | 32.738 | 97.084 | 77.587 | 121.299 | 0.308362 |
| PDE5A | 23.207 | 12.116 | 4.478 | 10.022 | 19.412 | 2.05299 |
| PDGFA | 28.927 | 15.237 | 5.801 | 18.04 | 18.729 | 2.038548 |
| PDK4 | 46.738 | 58.072 | 186.23 | 84.519 | 145.003 | 0.337254 |
| PDZK1IP1 | 24.767 | 12.483 | 1.526 | 8.185 | 9.95 | 3.779106 |
| PELI1 | 78.005 | 91.117 | 223.68 | 128.95 | 121.933 | 0.493117 |
| PER1 | 45.568 | 42.591 | 154.785 | 105.732 | 54.285 | 0.434254 |
| PF4 | 398.347 | 188.353 | 42.945 | 182.484 | 68.331 | 4.068086 |
| PILRA | 66.37 | 67.986 | 205.057 | 132.875 | 98.815 | 0.455893 |
| PITPNM2 | 31.267 | 24.171 | 8.955 | 16.87 | 19.802 | 2.055822 |
| PLA2G7 | 8.126 | 12.667 | 32.565 | 19.292 | 31.751 | 0.291575 |
| PLAGL1 | 7.28 | 6.915 | 19.335 | 12.527 | 13.169 | 0.484999 |
| PLD4 | 36.077 | 63.947 | 112.756 | 66.73 | 39.653 | 0.493892 |
| PLEK | 47.973 | 66.823 | 109.194 | 78.589 | 108.715 | 0.485396 |
| PMAIP1 | 6.63 | 5.997 | 22.694 | 20.044 | 13.803 | 0.35178 |
| POU5F1P3 | 10.141 | 16.216 | 30.428 | 29.147 | 8.877 | 0.444443 |
| PPBP | 2098.668 | 968.262 | 258.382 | 743.882 | 1004.435 | 3.137493 |
| PPP2R5C | 31.332 | 44.243 | 32.26 | 42.677 | 122.616 | 0.475801 |
| PRKAR2B | 208.079 | 97.909 | 33.684 | 66.145 | 148.319 | 2.515583 |
| PRKRA | 16.836 | 36.288 | 27.985 | 42.259 | 33.8 | 0.485448 |
| PROS1 | 16.446 | 7.343 | 3.155 | 4.844 | 13.656 | 2.278365 |
| PTGS1 | 137.549 | 61.683 | 26.459 | 21.881 | 72.575 | 3.412703 |
| PTK2 | 35.557 | 28.394 | 16.181 | 15.367 | 16.534 | 2.218523 |
| QKI | 39.783 | 41.489 | 84.974 | 57.626 | 141.296 | 0.420397 |
| QPCT | 12.091 | 13.095 | 34.702 | 16.954 | 24.387 | 0.477006 |
| RAB43 | 110.248 | 103.784 | 52.409 | 62.721 | 46.53 | 2.045924 |
| RAD23B | 76.185 | 52.198 | 44.573 | 37.415 | 29.118 | 2.05709 |
| RANBP17 | 19.631 | 9.669 | 10.38 | 4.677 | 3.999 | 3.090523 |
| RASGRF2 | 26.067 | 16.767 | 5.292 | 12.862 | 18.875 | 2.111885 |
| RBPJ | 30.812 | 34.023 | 56.378 | 51.446 | 115.446 | 0.41401 |
| RCBTB1 | 22.622 | 26.436 | 32.463 | 40.088 | 63.259 | 0.499713 |
| RELT | 8.776 | 11.872 | 33.074 | 16.119 | 8.682 | 0.454911 |
| REXO4 | 86.001 | 89.954 | 35.313 | 43.93 | 26.386 | 2.442539 |
| RNU12 | 22.362 | 49.995 | 78.054 | 95.125 | 37.263 | 0.318786 |
| RORC | 19.956 | 10.77 | 1.73 | 5.178 | 8.243 | 3.951422 |
| RPL13AP6 | 11.311 | 14.686 | 35.414 | 43.095 | 18.924 | 0.34827 |
| RPL29P2 | 7.346 | 9.24 | 19.132 | 42.176 | 5.56 | 0.329575 |
| RPS26 | 31.007 | 48.22 | 39.79 | 516.299 | 22.192 | 0.160858 |
| RSAD2 | 6.565 | 6.792 | 14.044 | 10.69 | 31.459 | 0.350488 |
| RUFY1 | 281.794 | 122.509 | 90.469 | 112.914 | 107.204 | 2.721885 |
| RUSC1 | 5.46 | 10.709 | 10.889 | 14.615 | 14.681 | 0.407615 |
| S100A11 | 460.166 | 475.105 | 1212.125 | 777.623 | 1650.876 | 0.379193 |
| S100A8 | 146.52 | 194.534 | 565.814 | 250.633 | 109.886 | 0.474516 |
| SAMD9L | 7.541 | 8.873 | 9.769 | 23.468 | 45.408 | 0.28766 |
| SAP30 | 10.986 | 15.054 | 28.596 | 35.411 | 23.85 | 0.375132 |
| SCARB2 | 12.481 | 12.667 | 21.371 | 15.284 | 43.75 | 0.46568 |
| SCARNA10 | 686.252 | 672.025 | 469.951 | 424.013 | 114.617 | 2.04124 |
| SCARNA16 | 382.096 | 280.694 | 263.775 | 225.328 | 41.457 | 2.160525 |
| SCP2 | 19.891 | 15.421 | 51.493 | 45.433 | 26.338 | 0.484107 |
| SDK2 | 39.523 | 36.716 | 10.177 | 29.481 | 4.78 | 2.668189 |
| SDPR | 704.063 | 310.189 | 73.067 | 209.627 | 218.992 | 4.210181 |
| SECTM1 | 26.457 | 31.453 | 83.447 | 40.004 | 60.674 | 0.431071 |
| SELP | 20.151 | 10.342 | 2.341 | 3.341 | 14.583 | 2.983124 |
| SERPINH1 | 17.291 | 18.542 | 6.208 | 12.277 | 5.999 | 2.118649 |
| SF3B4 | 9.036 | 14.748 | 37.755 | 18.708 | 35.604 | 0.294438 |
| SH3BGRL2 | 97.897 | 53.728 | 11.092 | 41.508 | 90.426 | 2.05341 |
| SIGLEC10 | 12.416 | 19.337 | 31.445 | 31.486 | 24.192 | 0.427533 |
| SIGLEC14 | 24.572 | 32.127 | 152.037 | 98.8 | 64.82 | 0.233532 |
| SIT1 | 75.275 | 78.144 | 18.521 | 38.084 | 47.505 | 2.1691 |
| SLA2 | 70.075 | 53.973 | 20.149 | 32.655 | 36.19 | 2.362238 |
| SLC11A2 | 73.065 | 50.117 | 38.569 | 13.112 | 18.436 | 3.126132 |
| SLC24A4 | 12.871 | 10.097 | 45.896 | 21.38 | 15.168 | 0.468354 |
| SLC29A1 | 5.655 | 5.446 | 20.251 | 10.356 | 7.901 | 0.440558 |
| SLC2A11 | 29.317 | 27.537 | 10.685 | 18.29 | 14.388 | 2.02825 |
| SLC35E2 | 24.182 | 23.376 | 63.501 | 58.545 | 70.672 | 0.376436 |
| SLC46A2 | 14.691 | 16.339 | 45.794 | 19.71 | 27.557 | 0.473593 |
| SLCO3A1 | 32.567 | 37.817 | 82.328 | 58.295 | 70.819 | 0.46207 |
| SMAD1 | 9.296 | 6.792 | 14.247 | 41.758 | 10.828 | 0.417279 |
| SMOX | 35.947 | 16.767 | 7.225 | 8.018 | 10.096 | 4.25593 |
| SMPD1 | 45.243 | 40.265 | 22.083 | 21.297 | 15.705 | 2.297182 |
| SMS | 19.111 | 32.922 | 46.303 | 35.495 | 75.598 | 0.36426 |
| SNORA14A | 19.176 | 8.322 | 4.478 | 8.602 | 3.609 | 3.447061 |
| SNORA14B | 159.326 | 71.78 | 31.954 | 35.996 | 22.338 | 5.293926 |
| SNORA16A | 1164.879 | 594.738 | 298.375 | 425.6 | 151.831 | 3.990195 |
| SNORA19 | 13.976 | 34.819 | 48.644 | 58.211 | 5.56 | 0.372975 |
| SNORA21 | 530.436 | 236.023 | 214.216 | 280.95 | 117.348 | 2.597994 |
| SNORA2A | 52.394 | 39.286 | 30.326 | 40.004 | 6.536 | 2.044883 |
| SNORA2B | 7.02 | 11.933 | 14.451 | 26.725 | 3.268 | 0.473855 |
| SNORA31 | 202.359 | 119.817 | 70.523 | 87.442 | 59.698 | 2.789068 |
| SNORA50 | 660.965 | 561.51 | 351.599 | 450.321 | 161.342 | 2.058521 |
| SNORA53 | 359.084 | 1812.791 | 1546.423 | 1787.672 | 423.4 | 0.286694 |
| SNORA57 | 41.148 | 38.613 | 15.672 | 21.297 | 3.609 | 3.042141 |
| SNORA5C | 120.323 | 132.851 | 60.856 | 83.517 | 25.801 | 2.121176 |
| SNORA62 | 196.378 | 159.837 | 78.257 | 118.51 | 53.455 | 2.354445 |
| SNORA66 | 251.437 | 152.249 | 127.105 | 171.626 | 66.917 | 2.062943 |
| SNORA70G | 83.596 | 143.743 | 185.314 | 305.336 | 115.983 | 0.41341 |
| SNORA74A | 86.651 | 60.275 | 39.485 | 36.998 | 44.286 | 2.152481 |
| SNORA9 | 777.518 | 876.656 | 481.858 | 553.631 | 23.509 | 2.202605 |
| SNORD15A | 352.324 | 217.665 | 153.869 | 171.877 | 163.146 | 2.161974 |
| SNPH | 16.901 | 16.277 | 4.376 | 14.782 | 3.512 | 2.236568 |
| SNX10 | 8.191 | 8.812 | 23.915 | 13.112 | 16.534 | 0.458785 |
| SNX19 | 21.971 | 24.967 | 59.94 | 50.528 | 42.725 | 0.430261 |
| SPARC | 498.649 | 303.335 | 73.271 | 187.161 | 320.976 | 2.572973 |
| SRP72 | 52.004 | 43.508 | 80.7 | 87.275 | 146.612 | 0.495926 |
| STEAP4 | 17.486 | 13.157 | 47.524 | 19.292 | 46.969 | 0.461027 |
| STK17A | 118.178 | 116.451 | 39.485 | 42.677 | 58.723 | 2.516478 |
| STK32C | 14.301 | 15.176 | 54.851 | 33.574 | 18.144 | 0.402584 |
| STMN3 | 76.12 | 73.126 | 20.251 | 45.015 | 29.264 | 2.415741 |
| SYNJ2 | 56.359 | 40.326 | 20.455 | 20.796 | 41.067 | 2.053949 |
| TAF4B | 14.366 | 10.831 | 3.867 | 5.429 | 8.389 | 2.43698 |
| TAS2R41 | 7.346 | 16.094 | 8.243 | 30.149 | 7.56 | 0.479587 |
| TBC1D8 | 7.671 | 15.788 | 27.985 | 21.547 | 21.363 | 0.324607 |
| TBX21 | 32.307 | 46.568 | 27.07 | 61.468 | 138.223 | 0.427415 |
| TCEA3 | 140.8 | 127.221 | 47.321 | 83.099 | 26.972 | 2.683745 |
| TCF4 | 7.15 | 11.749 | 23.813 | 8.185 | 11.267 | 0.495782 |
| TCF7L2 | 12.546 | 18.113 | 52.307 | 38.835 | 42.335 | 0.281981 |
| TGFBR3 | 38.418 | 52.565 | 36.127 | 48.106 | 159.586 | 0.472703 |
| THBS1 | 79.045 | 45.773 | 29.614 | 30.651 | 35.653 | 2.472268 |
| TLR1 | 33.867 | 35.125 | 47.423 | 59.38 | 123.201 | 0.441736 |
| TMEM170B | 26.262 | 29.312 | 128.529 | 52.281 | 85.646 | 0.295681 |
| TMEM176B | 124.939 | 121.53 | 100.849 | 10.774 | 60.04 | 2.183447 |
| TMEM40 | 31.787 | 23.253 | 7.531 | 17.121 | 6.536 | 3.057618 |
| TNFAIP2 | 39.263 | 65.66 | 124.459 | 73.411 | 60.479 | 0.45593 |
| TNFSF13B | 25.157 | 27.721 | 82.43 | 34.91 | 56.577 | 0.433948 |
| TPM1 | 33.802 | 16.645 | 5.801 | 7.516 | 16.095 | 3.447776 |
| TRAPPC5 | 29.707 | 24.661 | 3.969 | 10.106 | 13.169 | 3.271216 |
| TREM1 | 11.441 | 12.055 | 54.953 | 22.215 | 19.558 | 0.354848 |
| TREML2 | 46.933 | 33.167 | 11.398 | 16.035 | 23.362 | 2.771907 |
| TRIM58 | 75.99 | 37.206 | 9.769 | 25.055 | 58.625 | 2.439512 |
| TSEN2 | 92.696 | 81.265 | 58.413 | 23.635 | 23.996 | 2.622383 |
| TTC30A | 5.655 | 6.548 | 9.261 | 27.978 | 4.341 | 0.408009 |
| TUBA4A | 121.363 | 95.645 | 47.626 | 59.213 | 53.309 | 2.273453 |
| TUBA8 | 36.402 | 16.889 | 5.699 | 16.119 | 16.144 | 2.876719 |
| TUBB1 | 452.236 | 210.383 | 45.387 | 125.442 | 167.682 | 4.00787 |
| TYMP | 109.077 | 149.801 | 368.797 | 206.369 | 104.326 | 0.481582 |
| TYROBP | 1032.855 | 1316.269 | 3236.539 | 1872.942 | 1222.11 | 0.489382 |
| UBE2D2 | 716.544 | 590.454 | 372.257 | 261.073 | 191.045 | 2.60759 |
| UBE3A | 451.001 | 453.381 | 247.289 | 172.712 | 90.474 | 2.650478 |
| UBLCP1 | 24.312 | 20.439 | 25.441 | 19.793 | 104.814 | 0.486084 |
| UPK3B | 7.346 | 10.77 | 5.088 | 46.018 | 1.658 | 0.417671 |
| URI1 | 413.363 | 383.376 | 187.451 | 160.268 | 138.906 | 2.548346 |
| USP48 | 33.737 | 68.72 | 89.96 | 67.732 | 47.847 | 0.492417 |
| USP53 | 160.496 | 121.102 | 33.888 | 69.486 | 75.891 | 2.685901 |
| VAMP2 | 133.454 | 163.57 | 222.255 | 264.497 | 447.104 | 0.428719 |
| VCL | 36.793 | 14.074 | 6.818 | 13.864 | 27.411 | 2.295116 |
| VIM | 858.513 | 1560.92 | 3102.209 | 2469.25 | 1308.195 | 0.37437 |
| VNN1 | 8.906 | 6.425 | 27.273 | 18.123 | 22.777 | 0.391915 |
| VPS29 | 44.658 | 59.419 | 119.879 | 92.035 | 75.696 | 0.465818 |
| WARS | 80.605 | 121.408 | 247.391 | 125.442 | 204.75 | 0.418667 |
| XAF1 | 65.914 | 63.825 | 89.35 | 87.442 | 243.72 | 0.470241 |
| ZBTB16 | 31.657 | 35.125 | 12.212 | 13.279 | 18.729 | 2.147693 |
| ZBTB45 | 19.956 | 16.583 | 6.716 | 9.771 | 7.56 | 2.489624 |
| ZFHX3 | 19.241 | 20.316 | 49.661 | 29.314 | 50.188 | 0.4469 |
| ZFP36 | 283.679 | 220.48 | 570.088 | 534.756 | 729.013 | 0.464069 |
| ZNF259 | 8.971 | 11.26 | 17.809 | 17.455 | 20.29 | 0.484448 |
| ZNF385A | 28.602 | 31.025 | 88.23 | 30.4 | 87.938 | 0.415389 |
| ZNF569 | 42.318 | 43.998 | 20.149 | 20.712 | 16.193 | 2.225155 |
| ZNF683 | 8.061 | 13.952 | 13.535 | 43.846 | 10.779 | 0.354798 |
| ZNF93 | 25.872 | 18.48 | 16.995 | 9.103 | 8.925 | 2.216144 |

Table S4: Upregulated genes in KEGG Rap1 signaling pathway, as determined by DAVID Functional Annotation.

| Symbol | Pre-treatment | post-treatment | control 1 | control 2 | control 3 | Change vs. control mean |
| --- | --- | --- | --- | --- | --- | --- |
| ADCY3 | 33.607 | 33.167 | 14.959 | 16.703 | 18.046 | 2.028265 |
| ADORA2A | 17.031 | 21.234 | 6.309 | 12.11 | 7.072 | 2.004354 |
| FLT4 | 13.586 | 10.342 | 2.646 | 7.767 | 7.462 | 2.280168 |
| ITGA2B | 156.336 | 70.862 | 10.38 | 24.721 | 54.724 | 5.221353 |
| ITGB3 | 476.677 | 284.916 | 45.998 | 70.989 | 198.068 | 4.538988 |
| MAPK13 | 60.519 | 49.567 | 21.065 | 39.67 | 26.972 | 2.07004 |
| PDGFA | 28.927 | 15.237 | 5.801 | 18.04 | 18.729 | 2.038548 |
| THBS1 | 79.045 | 45.773 | 29.614 | 30.651 | 35.653 | 2.472268 |
